# Assessment of drought tolerance of 49 switchgrass (*Panicum virgatum*) genotypes using physiological and morphological parameters

**DOI:** 10.1186/s13068-015-0342-8

**Published:** 2015-09-22

**Authors:** Yiming Liu, Xunzhong Zhang, Hong Tran, Liang Shan, Jeongwoon Kim, Kevin Childs, Erik H. Ervin, Taylor Frazier, Bingyu Zhao

**Affiliations:** Department of Crop and Soil Environmental Science, Virginia Tech, 367 Smyth Hall, 185 Ag Quad Ln, Blacksburg, VA 24061 USA; Department of Statistics, Virginia Tech, Blacksburg, VA USA; Department of Plant Biology, Michigan State University, East Lansing, MI USA; Center for Genomics-Enabled Plant Science, Michigan State University, East Lansing, MI USA; Department of Horticulture, Virginia Tech, 407 Latham Hall, 220 Ag Quad Ln, Blacksburg, VA 24061 USA

**Keywords:** *Panicum virgatum*, Germplasm, Drought tolerance, SRAP marker, Genetic diversity, PCA, Metabolites

## Abstract

**Background:**

Switchgrass (*Panicum virgatum* L.) is a warm-season C_4_ grass that is a target lignocellulosic biofuel species. In many regions, drought stress is one of the major limiting factors for switchgrass growth. The objective of this study was to evaluate the drought tolerance of 49 switchgrass genotypes. The relative drought stress tolerance was determined based on a set of parameters including plant height, leaf length, leaf width, leaf sheath length, leaf relative water content (RWC), electrolyte leakage (EL), photosynthetic rate (Pn), stomatal conductance (*g*_s_), transpiration rate (Tr), intercellular CO_2_ concentration (Ci), and water use efficiency (WUE).

**Results:**

SRAP marker analysis determined that the selected 49 switchgrass genotypes represent a diverse genetic pool of switchgrass germplasm. Principal component analysis (PCA) and drought stress indexes (DSI) of each physiological parameter showed significant differences in the drought stress tolerance among the 49 genotypes. Heatmap and PCA data revealed that physiological parameters are more sensitive than morphological parameters in distinguishing the control and drought treatments. Metabolite profiling data found that under drought stress, the five best drought-tolerant genotypes tended to have higher levels of abscisic acid (ABA), spermine, trehalose, and fructose in comparison to the five most drought-sensitive genotypes.

**Conclusion:**

Based on PCA ranking value, the genotypes TEM-SEC, TEM-LoDorm, BN-13645-64, Alamo, BN-10860-61, BN-12323-69, TEM-SLC, T-2086, T-2100, T-2101, Caddo, and Blackwell-1 had relatively higher ranking values, indicating that they are more tolerant to drought. In contrast, the genotypes Grif Nebraska 28, Grenville-2, Central Iowa Germplasm, Cave-in-Rock, Dacotah, and Nebraska 28 were found to be relatively sensitive to drought stress. By analyzing physiological response parameters and different metabolic profiles, the methods utilized in this study identified drought-tolerant and drought-sensitive switchgrass genotypes. These results provide a foundation for future research directed at understanding the molecular mechanisms underlying switchgrass tolerance to drought.

**Electronic supplementary material:**

The online version of this article (doi:10.1186/s13068-015-0342-8) contains supplementary material, which is available to authorized users.

## Background

Switchgrass (*Panicum virgatum* L.) has been designated as a model bioenergy crop in the United States [1]. As a warm-season perennial grass native to North America, switchgrass produces substantial aboveground biomass and has adapted to grow over an extensive range of habitats [2, 3]. To avoid competition with food crops for arable land, switchgrass will primarily be grown on marginal land, of which millions of hectares are affected by drought [4]. Drought stress will be one of the major abiotic stresses encountered when growing switchgrass for use as a biofuel. Indeed, a recent study suggests that drought stress could be one major economic risk factor that limits biofuel production [5]. Therefore, a major goal of switchgrass breeding programs is to identify and select for genotypes with improved tolerance to drought stress [6].

Two distinct switchgrass ecotypes, lowland and upland, have been recognized and are generally defined based on their morphological characteristics and habitat preferences. Lowland ecotypes are mostly tetraploid (2*n* = 4× =36), whereas upland ecotypes tend to be octaploid (2*n* = 8× =72) with a few tetraploid exceptions [7]. In addition, lowland ecotypes are usually tall, coarse in leaf texture, and are adapted to grow in the flood plain region of North America. Alternatively, upland ecotypes are shorter, have finer leaves, and are predominantly found in the cooler climates of the northern United States [8, 9]. Previous studies have evaluated a number of switchgrass germplasm cultivars in response to drought stress [4, 10, 11]. Jiang et al. [4] found that drought stress in the upland switchgrass cultivar Cave-in-Rock reduced tissue water content and leaf dry weight while simultaneously increasing total carotenoid concentration and electrolyte leakage. Interestingly, the values of these parameters returned to those similar to the control (well-watered) plants after re-watering [4]. Under greenhouse conditions, Barney et al. [10] estimated that drought treatments (−4.0 and −11.0 MPa) could decrease the height and number of tillers, as well as decrease the overall leaf area, of drought-stressed switchgrass plants. They also found that drought treatments reduced biomass yields by up to 80 % [10]. In a field trial using the switchgrass cultivar Sunburst, drought stress reduced yields to approximately 26 % of those obtained in a year with above-average precipitation [12]. Although upland switchgrass genotypes have generally been considered to be more drought tolerant than lowland genotypes [13, 14], lowland switchgrass cultivars have been reported to outperform upland cultivars under various adverse environmental conditions, including drought stress [10]. Thus, a more systematic evaluation of drought tolerance, one that examines a greater number of diverse lowland and upland switchgrass cultivars in a controlled manner, is required.

It is difficult to assess drought stress tolerance of a large collection of switchgrass germplasm based solely on the data collected from a drought treatment experiment, because there is significant genetic and phenotypic variation among switchgrass germplasms under non-stressed (control) conditions. The Drought Stress Index (DSI) is a method to evaluate the effect of drought stress on individual germplasm based on the difference between drought treatment and the control plants. DSI is calculated as DSI = (value of trait under stress condition)/(value of trait under controlled condition) × 100. This equation removes the effect of germplasm variation from the drought stress evaluation and can therefore be used to assess a large collection of germplasm simultaneously [15].

Drought stress has a wide range of effects on the morphological, physiological, and biochemical processes in plants, and it can negatively affect the productivity of both dry land and irrigated crops [16–18]. Drought-tolerant plants usually possess a combination of distinct morphological and physiological characteristics such as reduced leaf area, an extensive root system, the ability to sustain high leaf tissue water potential, and maintenance of a higher chlorophyll content and photosynthetic efficiency under drought conditions [19, 20]. Physiological measurements such as leaf relative water content (RWC), electrolyte leakage (EL), photosynthetic rate (Pn), stomatal conductance (g_s_), transpiration rate (Tr), and water use efficiency (WUE) have been widely used as markers for evaluating drought stress tolerance in various plant species [21, 22]. Plant hormones such as abscisic acid (ABA) and jasmonic acid (JA) play an important role in plant response to drought stress [23]. An increase in JA is required for ABA levels to increase under drought conditions [24]. A recent study has shown that exogenous spraying of JA activates the plant antioxidant defense system and improves drought tolerance in some *Brassica* species [25]. Therefore, ABA and JA levels are routinely used as indicators of plant drought tolerance. In response to drought treatments, a variety of other metabolites are synthesized, including amino acids (e.g., proline) [26, 27], nonstructural carbohydrates (e.g., glucose, fructose, sucrose, raffinose, and trehalose), inositol and inositol-phosphates, polyamines (PAs) (e.g., putrescine, spermidine, and spermine), and glycine betaine (GB). Increased carbohydrate turnover has also been observed in drought-tolerant plants [27, 28]. Proline, sugars, and glycine betaine are osmotically neutral metabolites that play important roles in osmotic adjustment [29–32]. In guard cells, inositol phosphates can release vacuolar Ca^2+^ into the cytosol in response to drought stress [33]. Polyamines (PAs) are ubiquitous, nitrogen-containing polycationic compounds that are found in all eukaryotic cells. In plants, the most abundant PAs are putrescine, spermidine, and spermine, and an increase in PA levels has been closely correlated with drought tolerance [34–36]. Therefore, metabolic profiling of drought-stressed plants could help evaluate their tolerance to drought stress.

Various molecular markers have been used to evaluate the genetic diversity within and between switchgrass genotypes [37–39]. Among the different types of markers, sequence-related amplified polymorphism (SRAP) markers are useful because of their reproducibility, low cost, ability to amplify without prior knowledge of the target sequence, and ease of use [40]. SRAP markers have been successfully used to evaluate genetic diversity and to construct genetic maps in species ranging from field crops to forage grasses and tree species [40–43].

Systematically evaluating diverse switchgrass germplasms in response to drought stress will be helpful for identifying genetic resources that can be used to breed elite switchgrass cultivars with improved drought tolerance. Switchgrass germplasms with distinct responses to drought stress will be useful for studying the mechanisms underlying drought tolerance and for identifying genes or molecular markers that can be used for molecular breeding. The objectives of this study were: (1) to determine the morphological, physiological, and metabolic parameters that are important indicators of switchgrass drought tolerance, and (2) to identify drought-tolerant and drought-sensitive switchgrass genotypes from 49 genetically diverse lowland and upland switchgrass genotypes.

## Results

### UPGMA clustering analysis to evaluate the genetic background of 49 switchgrass genotypes

**Fig. 1:**
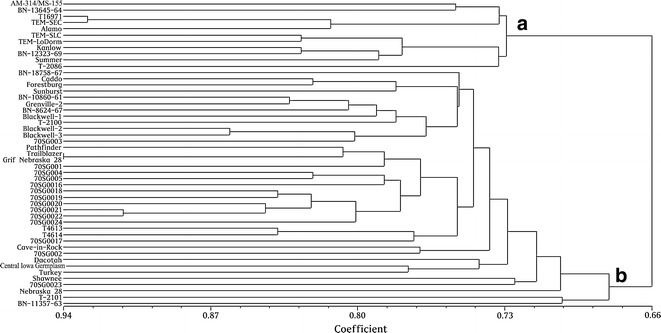
UPGMA dendrograms of cluster analysis of the 49 switchgrass genotypes based on the similarity coefficients calculated using SRAP data

Switchgrass has a diverse geographic distribution [8]. Presently, a method for efficient systematic evaluation of diverse switchgrass germplasms for drought tolerance has not yet been reported. In this study, we selected 49 switchgrass genotypes from 49 accessions that include both upland and lowland ecotypes for drought stress evaluation (Table 1). To estimate the genetic diversity of the 49 switchgrass genotypes, we performed SRAP analysis using 12 primer pairs (Table 2). The 12 SRAP primer pairs produced a total of 180 DNA markers, of which 167 were polymorphic (representing 92.4 % of all bands). The SRAP data were used for UPGMA cluster analysis (Fig. 1) at a genetic similarity coefficient value of 0.66. The results of the UPGMA cluster analysis revealed that the 49 genotypes clustered into two groups. Eleven genotypes (AM-314/MS-155, BN-13645-64, T16971, TEM-SEC, Alamo, TEM-SLC, TEM-LoDorm, Kanlow, BN-12323-69, Summer, and T-2086) diverged from the others and closely clustered into one group (cluster a). This group included all of the lowland genotypes used in this study (AM-314/MS-155, BN-13645-64, BN-11357-63, Alamo, TEM-SEC, TEM-SLC, TEM-LoDorm, T-2086, BN-12323-69, and Kanlow). Interestingly, T16971 and Summer, two upland genotypes, also clustered into the lowland group (cluster a). This could be attributed to the limited number of primers (12 pairs) used for SRAP analysis and/or the close genetic background of these upland and lowland genotypes. The other 38 genotypes that were evaluated in this study clustered together in a second large group (cluster b). Thus, the results of SRAP analysis revealed that the selected 49 genotypes represent a diverse genetic pool of switchgrass germplasm.

**Table 1:** List of switchgrass accessions

Genotype no.	Accession	Plantid	Ecotype	Genotype no.	Accession	Plantid	Ecotype
1	PI 421999	AM-314/MS-155	Lowland	26	PI 414066	Grenville-2	Upland
2	PI 315728	BN-13645-64	Lowland	27	PI 476292	T-2100	Upland
3	PI 422006	Alamo	Lowland	28	Grif 16407	Blackwell-1	Upland
4	PI 607838	TEM-SEC	Lowland	29	Grif 16409	Blackwell-2	Upland
5	PI 607837	TEM-SLC	Lowland	30	PI 421520	Blackwell-3	Upland
6	PI 636468	TEM-LoDorm	Lowland	31	PI 642192	Pathfinder	Upland
7	PI 421521	Kanlow	Lowland	32	PI 549094	Trailblazer	Upland
8	PI 476296	T16971	Upland	33	Grif 16408	Grif Nebraska 28	Upland
9	PI 414070	BN-12323-69	Lowland	34	Grif 16054	Central Iowa Germplasm	Upland
10	PI 414068	BN-18758-67	Upland	35	PI 204907	Turkey	Upland
11	PI 476290	T-2086	Lowland	36	PI 642193	70SG001	Upland
12	PI 476293	T-2101	Upland	37	PI 642194	70SG002	Upland
13	PI 315727	BN-11357-63	Lowland	38	PI 642195	70SG003	Upland
14	PI 642191	Summer	Upland	39	PI 642196	70SG004	Upland
15	PI 469228	Cave-in-Rock	Upland	40	PI 642197	70SG005	Upland
16	PI 591824	Shawnee	Upland	41	PI 642207	70SG0016	Upland
17	PI 476297	Caddo	Upland	42	PI 642208	70SG0017	Upland
18	PI 478001	Forestburg	Upland	43	PI 642209	70SG0018	Upland
19	PI 598136	Sunburst	Upland	44	PI 642210	70SG0019	Upland
20	PI 477003	Nebraska 28	Upland	45	PI 642211	70SG0020	Upland
21	PI 537588	Dacotah	Upland	46	PI 642212	70SG0021	Upland
22	PI 476295	T4613	Upland	47	PI 642213	70SG0022	Upland
23	PI 476294	T4614	Upland	48	PI 642214	70SG0023	Upland
24	PI 315724	BN-10860-61	Upland	49	PI 642215	70SG0024	Upland
25	PI 414067	BN-8624-67	Upland

**Table 2:** The number of SRAP fragments generated from 12 primer pair combinations in switchgrass

Primer pair	Total no. of bands	No. of polymorphic bands	Percent polymorphic bands (%)
me7 + em15	14	13	92.9
me1 + em19	14	14	100.0
me4 + em19	10	9	90.0
me12 + em5	9	9	100.0
me2 + em4	19	18	94.7
me7 + em4	11	9	81.8
me8 + em13	16	14	87.5
me9 + em13	18	17	94.4
me11 + em15	22	22	100.0
me12 + em9	16	16	100.0
me3 + em3	12	10	83.3
me5 + em19	19	16	84.2
Total	180	167	92.4

### Physiological and morphological evaluation of the drought responses of 49 switchgrass genotypes by heatmap and PCA methods

We evaluated the 49 switchgrass genotypes for their responses to drought treatment. Drought responses in both well-watered and drought-stressed plants were measured using seven physiological parameters (RWC, EL, Pn, g_s_, Tr, Ci, and WUE) and four morphological traits [plant height, leaf length (LL), leaf width (LW), and leaf sheath length (SL)]. Our results found that the effects of soil moisture regime and genotype, as well as the interaction between soil moisture and genotype, were significant (*p* ≤ 0.05) for all physiological parameters (Table 3). However, for each of the morphological parameters (plant height, LL, LW, and SL), the effects of soil moisture regime and the interaction between soil moisture and genotype were not significant (*p* ≤ 0.05) (Table 3).

**Table 3:** Summary of analysis of variance for the effects of treatments, lines, and the interaction on leaf relative water content (RWC), electrolyte leakage (EL), photosynthetic rate (Pn), stomatal conductance (g_s_), transpiration rate (Tr), intercellular CO_2_ concentration (Ci), water use efficiency (WUE), leaf length (LL), leaf width (LW) and leaf sheath length (SL) with the data of 30 days

Variable	Pn	EL	RWC	Tr	*g*_s_	WUE	Height	LL	LW	SL
Treatment	***	***	***	***	***	***	NS	NS	NS	*
Lines	**	**	**	*	*	*	***	**	*	**
Treatment × lines	*	*	*	*	*	NS	NS	NS	NS	NS

** Significant at *P* ≤ 0.01, *** significant at *P* ≤ 0.001, *NS* nonsignificant at *P* ≤ 0.05

**Fig. 2:**
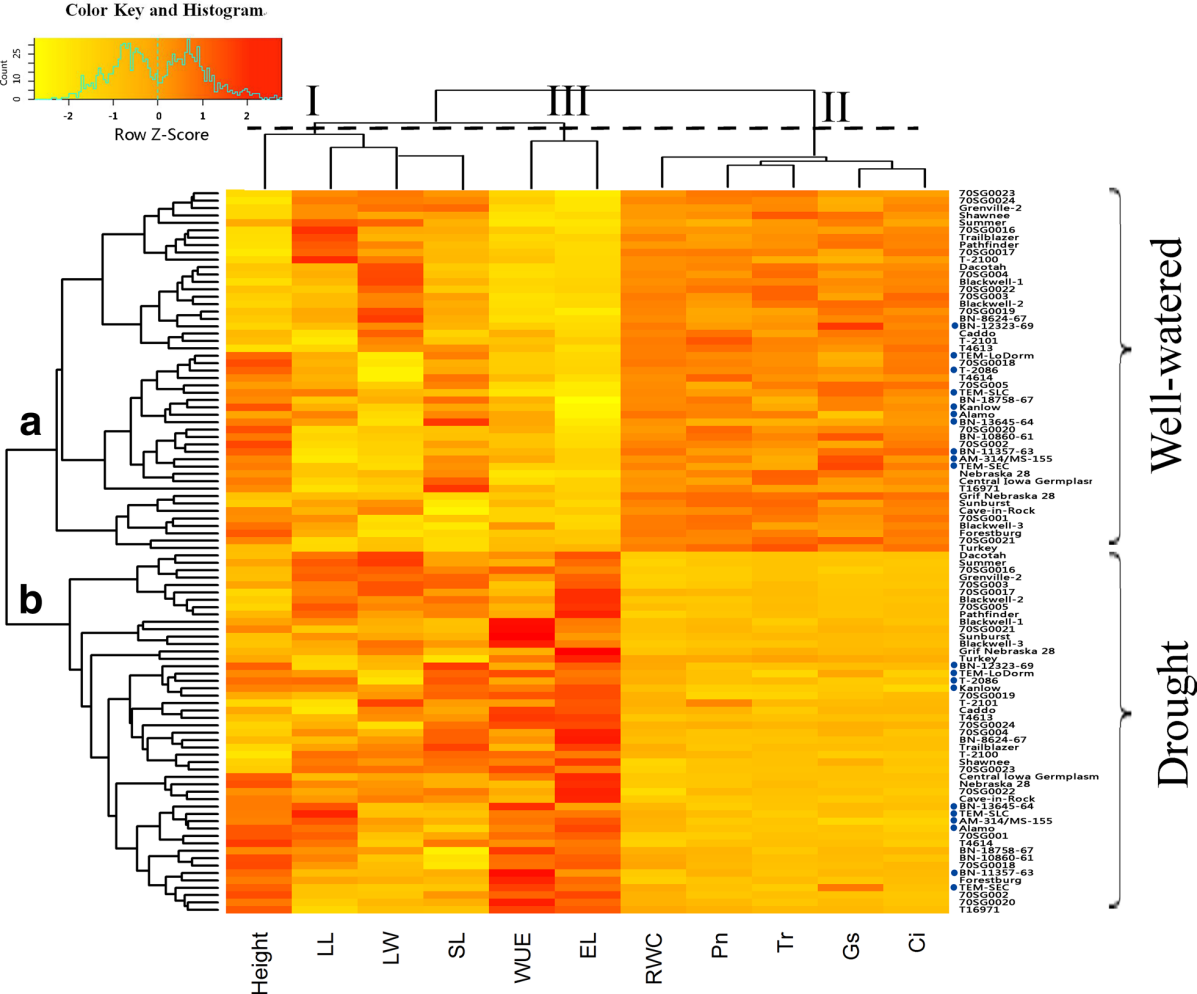
Heatmap and hierarchical clustering for morphological and physiological parameters under well-watered and drought stress conditions in 49 switchgrass genotypes after 30 days of treatment. Clustering analysis of switchgrass genotypes (*left*) showed two main groups where the group a represents 49 genotypes under the well-watered condition; while group b represents those genotypes under the drought treatment. The clustering analysis of different parameters (*top*) showed three major groups: group I includes all morphological parameters, group II include the other five physiological parameters, while group III include two key physiological parameters associate with drought tolerance. *RWC* relative water content, *EL* electrolyte leakage, *Pn* photosynthetic rate, *g*_*s*_ stomatal conductance, *Tr* transpiration rate, *Ci* intercellular CO_2_ concentration, *WUE* water use efficiency, *LL* leaf length, *LW* leaf width, *SL* leaf sheath length

To identify the key parameters for assessing drought tolerance in switchgrass, both physiological and morphological measurements were used to plot a heatmap. As shown in Fig. 2, the morphological and physiological measurements of the 49 genotypes, grown under either drought treatment or well-watered conditions (control), were used for hierarchical (row) clustering. When grown under well-watered conditions, the 49 genotypes clustered into group a while the same set of 49 genotypes grown under drought conditions clustered into group b. This clear clustering demonstrates that in comparison to control conditions, drought stress treatment alters both the physiological and morphological characteristics for each switchgrass genotype. Interestingly, most of the lowland genotypes tended to cluster together under well-watered conditions (group a in Fig. 2, dot-highlighted); however, these genotypes are scattered under drought stress conditions (group b in Fig. 2, dot-highlighted).

**Fig. 3:**
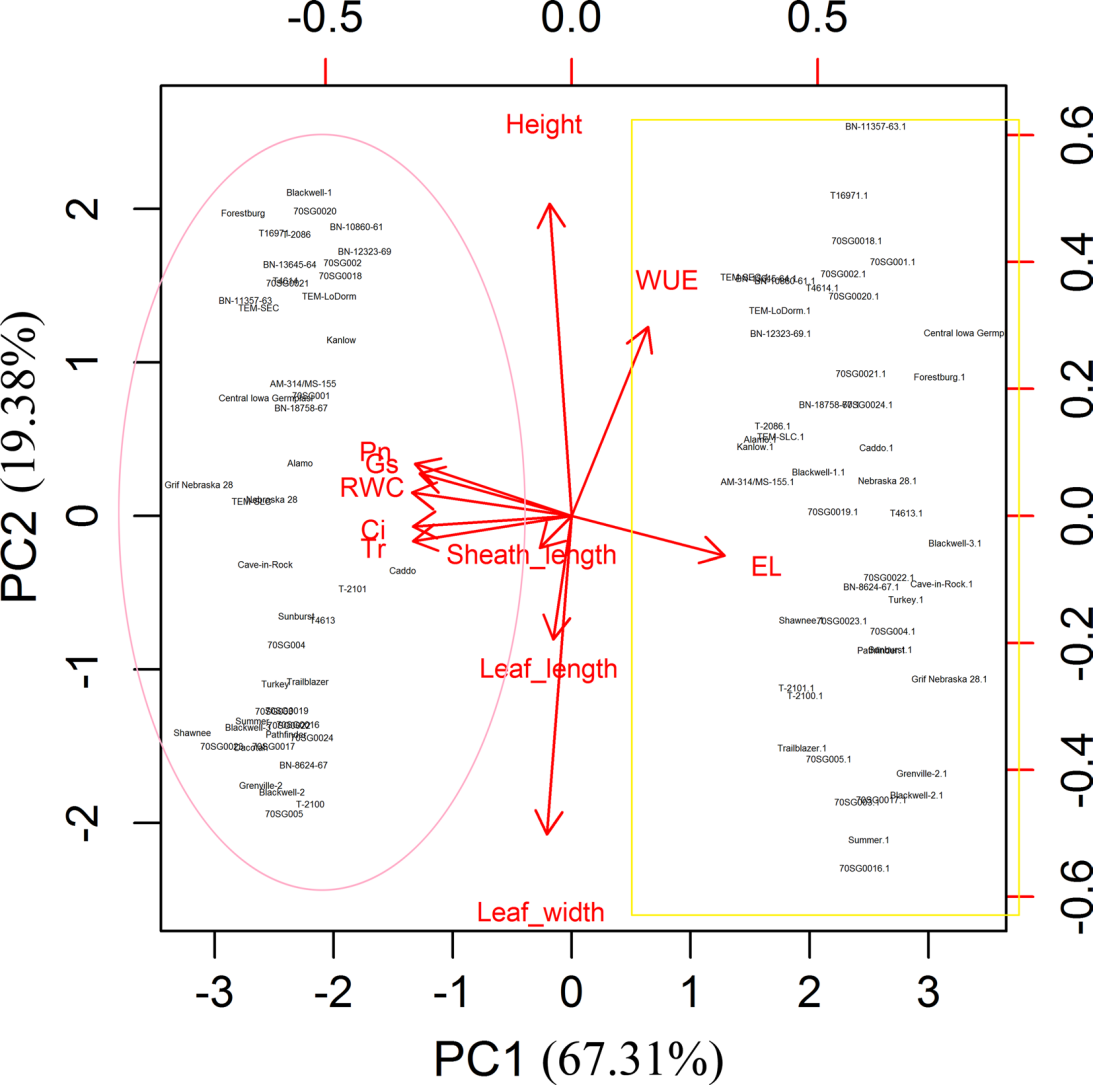
Principal component analysis biplot of morphological and physiological traits of 49 switchgrass genotypes under well-watered and drought stress conditions after 30 days of treatment. The seven physiological parameters (Pn, Ci, *g*_s_, Tr, RWC, WUE and EL) allow to separate 49 switchgrass genotypes that were either grown under well-watered (*circled*) or drought treatment (*box*) conditions. *Arrows* represent physiological traits with various length based on the impact of each trait on the separation of genotypes. *RWC* relative water content, *EL* electrolyte leakage, *Pn* photosynthetic rate, *g*_*s*_ stomatal conductance, *Tr* transpiration rate, *Ci* intercellular CO_2_ concentration, *WUE* water use efficiency, *LL* leaf length, *LW* leaf width, *SL* leaf sheath length

To evaluate the contributions of each parameter in the control and drought-treated switchgrass plants, we performed PCA using both physiological (RWC, EL, Pn, g_s_, Tr, Ci, and WUE) and morphological (plant height, LL, LW, and SL) parameters collected from plants after 30 days of drought treatment. The physiological parameters contributed more than the morphological parameters to the separation of the control and drought-treated groups (Fig. 3). Among the seven physiological parameters, Pn, g_s_, Ci, Tr, and RWC were positively associated with the control treatment (well-watered) group (Fig. 3, circled). WUE and EL were positively correlated with drought treatment (Fig. 3, box). The four morphological traits (plant height, LL, LW, and SL) did not contribute to the separation of the genotypes under either condition. A similar result was found after 15 days of drought treatment (Additional file 1: Figure S1).

Hierarchical clustering analysis of the heatmap also indicated that the physiological and morphological measurements could cluster the 49 genotypes into three distinct groups (top of Fig. 2 group I, II, III). The four morphological measurements, which reflect relative long-term response to abiotic stress, were clustered together (top of Fig. 2, group I) and were not consistently different between the control (Fig. 2, group a) and the short-term drought treatment groups (Fig. 2, group b). Thus, morphological traits do not appear to closely correlate with short-term drought tolerance in switchgrass (Additional file 2: Tables S1, S2).

The four photosynthesis-related traits (Pn, Tr, g_s_, and Ci) and RWC were clustered into group II, where all 49 genotypes showed decreased Pn, Tr, g_s_, Ci, and RWC under drought treatment (Fig. 2). When the 49 genotypes were evaluated using the drought stress index (DSI), significant differences in the DSI of Pn were observed among the 49 genotypes (Additional file 3: Figure S2). TEM-LoDorm, T2101, TEM-SEC, TEM-SLC, and Alamo all had a higher Pn than the other genotypes under drought stress, resulting in a comparatively higher DSI (>52.0 %). The upland genotypes, including Central Iowa Germplasm, 70SG001, Dacotah, Nebraska 28, and Grenville-2, had a lower DSI for Pn (<17.2 %) in comparison to the other genotypes. Drought stress reduced *g*_s_ in all 49 genotypes (Additional file 4: Figure S3). The BN-13645-64, TEM-SEC, 70SG0024, BN-10860-61, and TEM-LoDorm genotypes had DSIs for *g*_s_ of less than 44.5 %. Upland genotypes, including 70SG003, Grif Nebraska 28, Cave-in-Rock, Nebraska 28, and 70SG0016, had somewhat lower DSIs for *g*_s_, resulting in DSIs less than 14.6 %. There were significant differences in the DSI for Tr among the 49 genotypes (Additional file 5: Figure S4). BN-12323-69, TEM-SEC, BN-10860-61, BN-13645-64, and T-2100 had greater DSIs for Tr than the other genotypes. Overall, the Tr DSIs for these genotypes were greater than 45.9 %. Alternatively, Grenville-2, Dacotah, Blackwell-3, Central Iowa Germplasm, and 70SG0021 had lower DSI Tr values with DSIs less than 15.3 %. There were also significant differences in the DSIs for Ci among the 49 genotypes (Additional file 6: Figure S5). BN-18758-67, Caddo, BN-13645-64, 70SG0019, and TEM-LoDorm had comparatively higher DSIs for Ci, which resulted in DSIs greater than 48.4 %. In contrast, 70SG003, Nebraska 28, Grenville-2, Central Iowa Germplasm, and 70SG0016 had relatively lower DSIs for Ci, resulting in DSIs of less than 27.2 % for these genotypes. Drought stress reduced the RWC of all switchgrass genotypes (Additional file 7: Figure S6). The genotypes TEM-LoDorm, BN-12323-69, Alamo, TEM-SEC, and BN-10860-61 all had a DSI higher than 70.6 % for RWC. Conversely, the upland genotypes 70SG003, Grif Nebraska 28, 70SG0022, Grenville-2, and Summer all had DSIs lower than 39.3 % for RWC.

The WUE and EL were clustered into group III (top of Fig. 2). In general, all 49 genotypes showed increased WUE and EL under drought treatment. The WUE is a parameter that is derived from the Pn and Tr values. WUE consistently increased under drought treatment in all 49 genotypes, while the EL, a measurement of the damage of cell membrane, consistently increased in all 49 genotypes in response to drought treatment. As shown in Additional file 8: Figure S7, a large variation in WUE was observed. The genotypes Blackwell-3, Forestburg, 70SG0021, Sunburst, and BN-11357-63 tended to have higher DSIs (>187.7 %) for WUE. Alternatively, the genotypes 70SG0017, Grif Nebraska 28, 70SG003, BN-12323-69, and Pathfinder tended to have relatively lower DSIs (<96.5 %) for WUE. Several lowland genotypes, including BN-13645-64, Alamo, and TEM-SLC, had intermediate WUEs and DSIs ranging from 122.7 to 154.5 %. The EL reflects cell membrane damage that occurs during drought stress. In addition, the EL may also affect Tr and Pn (and subsequently affect WUE). Drought stress resulted in an increased EL for all genotypes (Additional file 9: Figure S8). The genotypes TEM-SEC, T4614, BN-11357-63, BN-13645-64, and T-2086 all had relatively lower DSIs (<173.7 %) for the EL values in comparison to the other genotypes. Overall, all 49 genotypes showed increased WUE and EL under drought treatment.

### The 49 switchgrass genotypes can be clustered into three groups based on DSI values for seven physiological measurements at 30 days of drought treatment

**Fig. 4:**
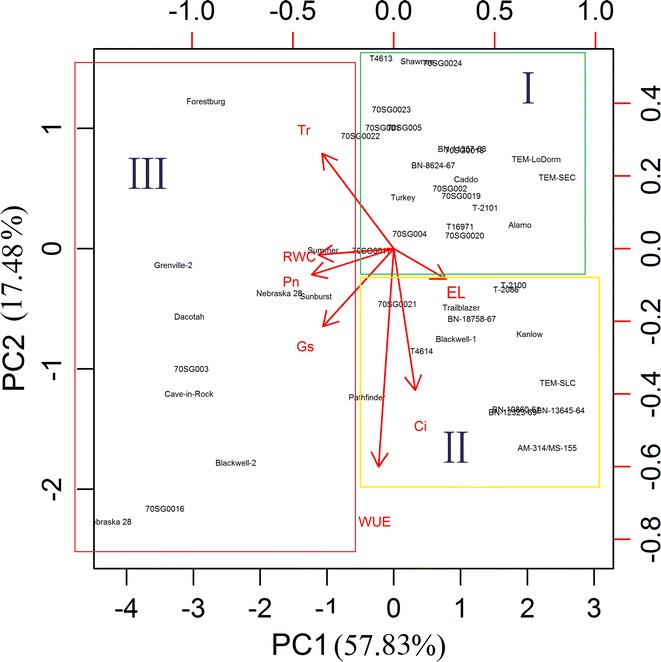
Principal component analysis biplot of the DSI of seven physiological traits of 49 switchgrass genotypes under well-watered (control) and drought stress conditions after 30 days of treatment. *Arrows* represent physiological traits with various length based on the impact of each trait on the separation of genotypes. The 49 switchgrass genotypes were clustered into three major groups. Group I include switchgrass genotypes that have the best performance based on DSI of the physiological parameters, while group III genotypes have the worst performance, and group II include the intermediate genotypes. The proportion of variance for principal component analysis based on the DSI of seven physiological traits is shown in Additional file 10: Figure S9, where it suggested the top two PCs can explain 75.31 % of total variation. *RWC* relative water content, *EL* electrolyte leakage, *Pn* photosynthetic rate, *g*_*s*_ stomatal conductance, *Tr* transpiration rate, *Ci* intercellular CO_2_ concentration, *WUE* water use efficiency

Heatmap hierarchical clustering and PCA indicated that physiological parameters are important for distinguishing the control and drought treatments in switchgrass. To cluster the switchgrass genotypes that had similar physiological responses to drought, we performed PCA using the DSI of seven physiological measurements collected at 30 days of drought treatment. The results of this PCA analysis identified three major groups (group I, II and III) (Fig. 4). In general, the lowland genotypes clustered mainly into groups I and II; however, upland genotypes such as BN-10860-61, T-2100, T-2101, Caddo, and BN-18758-67 also clustered in groups I and II. This suggests that these upland genotypes have similar tolerance to drought as their lowland counterparts.

PCA using the DSI values of seven physiological measurements collected at 30 days also suggests that the first principal component (PC1) explained approximately 57.83 % of the variance in the data and that the second (PC2) and third components (PC3) explained an additional 17.48 and 12.22 % of the variance, respectively. Together, the three components (PC1, PC2, and PC3) could explain 87.53 % of the variance among the 49 genotypes (Additional file 10: Figure S9). Because of the importance of physiological parameters for distinguishing the control and drought treatments in switchgrass, the relationships among the seven physiological parameters were further analyzed. We performed correlation analysis using Pearson’s method. Table 4 shows that under drought stress, the correlations of Pn with EL, RWC, Tr, *g*_s,_ and WUE were significant (*p* < 0.05) because they had large correlation coefficients (*r*) of −0.549, 0.555, 0.766, 0.737 and 0.847, respectively. These findings reveal that these physiological indicators, particularly Pn, are important parameters for assessing tolerance to abiotic stresses, including drought.

**Table 4:** The correlation coefficient (*r*) between physiological measurements in 49 switchgrass genotypes under drought stress

	Pn	EL	RWC	Tr	*g*_s_	WUE
Pn	1	−0.549***	0.555**	0.766***	0.737***	0.847***
EL		1	−0.188	−0.309*	−0.369**	−0.456**
RWC			1	0.525**	0.536***	0.421**
Tr				1	0.674***	0.472**
*g*_s_					1	0.511**
WUE						1

*, **, and *** indicate significance at the 0.05, 0.01, and 0.001 levels, respectively (*n* = 49)

### Drought tolerance ranking of the 49 switchgrass genotypes using integrated PCA values

Since the PCA that was based on the DSI of seven physiological parameters (Fig. 4; Additional file 10: Figure S9) showed that three major components (PC1, PC2, and PC3) could explain 87.53 % of the variance in response to drought treatment (Additional file 10: Figure S9), we developed the following formulas based on the results of PCA: (1) PC1 = 0.926 × RWC + 0.957 × Pn + 0.938 × Tr + 0.907 × *g*_s_ + (−0.372) × Ci + (−0.109) × WUE + (−0.649) × EL; (2) PC2 = 0.0034 × RWC + 0.134 × Pn + (−0.205) × Tr + 0.191 × *g*_s_ + 0.5144 × Ci + 0.888 × WUE + (−0.2719) × EL; and (3) PC3 = 0.06428 × RWC + 0.0089 × Pn + 0.1597 × Tr + 0.224 × *g*_s_ + 0.752 × Ci + (−0.363) × WUE + 0.2787 × EL. In addition, a ranking value for each switchgrass genotype was calculated using a separate formula [(Ranking value = (57.83 % × PC1) + (17.48 % × PC2) + (12.22 % × PC3)] [44]. The 49 switchgrass genotypes were then ranked for relative drought tolerance. Genotypes TEM-SEC, TEM-LoDorm, BN-13645-64, Alamo, BN-10860-61, BN-12323-69, TEM-SLC, T-2086, T-2100, T-2101, Caddo, and Blackwell-1 had relatively higher ranking values, suggesting that they were more drought tolerant (Table 5). In contrast, genotypes including Grif Nebraska 28, Grenville-2, Central Iowa Germplasm, Cave-in-Rock, Dacotah, and Nebraska 28 had relatively lower ranking values and thus, were found to be more sensitive to drought stress.

**Table 5:** The three major components (PC1, PC2 and PC3) and PCA ranking values of the physiological parameters of 49 switchgrass lines after 30 days of drought stress

	PC1	PC2	PC3	Ranking	Numeric rank
TEM-SEC	87.49	107.57	11.85	70.85	1
TEM-LoDorm	63.25	104.99	13.33	56.56	2
BN-13645-64	69.79	71.30	26.84	56.10	3
Alamo	69.12	68.30	26.50	55.15	4
BN-10860-61	65.49	44.07	38.37	50.26	5
BN-12323-69	66.67	36.81	40.43	49.93	6
TEM-SLC	53.58	95.92	14.45	49.52	7
T-2086	58.96	63.42	26.55	48.43	8
T-2100	55.28	73.88	23.46	47.75	9
T-2101	47.80	62.32	28.42	42.01	10
Caddo	43.77	73.18	24.35	41.08	11
Blackwell-1	30.59	94.67	15.16	36.09	12
AM-314/MS-155	37.03	46.80	35.42	33.92	13
70SG0019	23.45	64.54	25.49	27.96	14
70SG002	20.92	66.75	27.10	27.08	15
70SG0018	16.78	88.39	15.72	27.08	16
T16971	19.83	68.11	25.15	26.45	17
70SG0020	18.83	62.76	31.20	25.67	18
Trailblazer	23.00	43.38	34.70	25.13	19
BN-18758-67	17.86	60.66	32.70	24.93	20
BN-11357-63	5.73	128.58	−7.83	24.83	21
70SG0024	11.93	77.78	27.98	23.91	22
Forestburg	−19.99	168.44	−20.14	15.42	23
T4614	6.53	49.47	24.46	15.41	24
Sunburst	−15.56	140.08	−7.26	14.60	25
Kanlow	1.36	45.30	33.07	12.75	26
Shawnee	−12.10	104.59	10.56	12.57	27
BN-8624-67	−7.92	71.94	28.49	11.48	28
Turkey	−16.46	72.55	21.53	5.79	29
70SG0023	−23.86	74.50	19.51	1.61	30
Pathfinder	−16.00	36.80	30.91	0.96	31
70SG005	−26.21	54.68	36.22	−1.17	32
T4613	−34.79	88.37	22.77	−1.89	33
70SG0021	−50.75	149.10	−12.89	−4.86	34
70SG004	−34.57	41.19	47.56	−6.98	35
Blackwell-3	−68.04	189.88	−33.62	−10.27	36
Summer	−42.97	54.36	30.46	−11.63	37
70SG001	−49.36	88.38	11.94	−11.64	38
70SG0022	−56.64	62.00	33.21	−17.86	39
70SG0016	−64.46	84.77	8.47	−21.43	40
70SG0017	−57.00	5.22	56.18	−25.19	41
70SG003	−58.65	23.10	34.46	−25.67	42
Blackwell-2	−65.66	49.15	29.41	−25.79	43
Grif Nebraska 28	−63.46	15.19	40.02	−29.15	44
Grenville-2	−83.49	59.64	22.98	−35.05	45
Central Iowa Germplasm	−96.63	103.35	8.59	−36.76	46
Cave-in-Rock	−85.49	34.09	34.85	−39.22	47
Dacotah	−101.37	86.86	16.36	−41.44	48
Nebraska 28	−102.90	17.87	44.40	−50.96	49

### Different metabolic responses of the five best drought-tolerant genotypes and the five most drought-sensitive genotypes

**Fig. 5:**
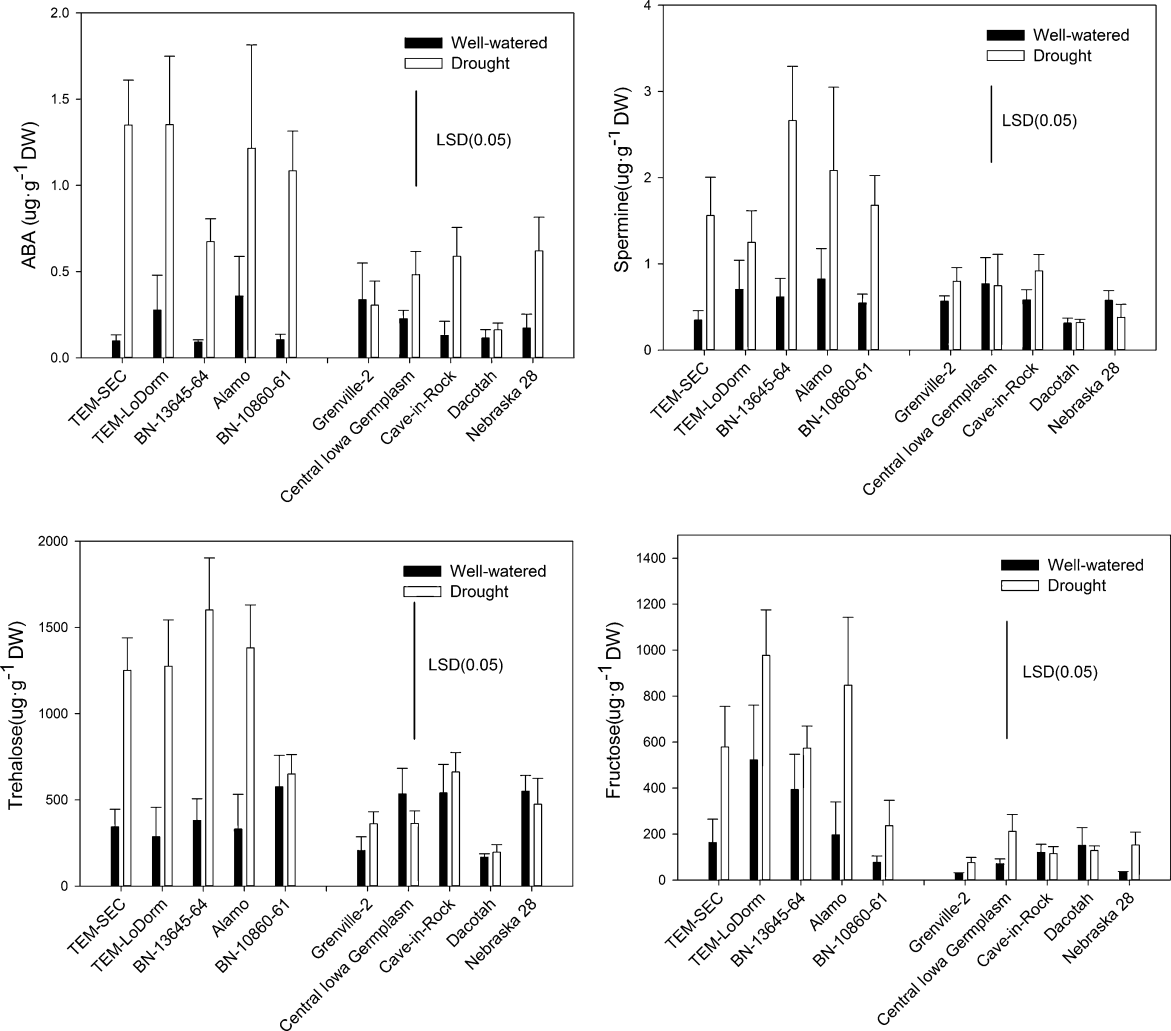
The levels of abscisic acid (ABA), spermine, trehalose, and fructose in five best drought-tolerant genotypes and the five most drought-sensitive genotypes under well-watered conditions (control, *solid bar*) and drought stress (*open bar*) after 30 days of treatment. The values are the means of six replicates (*n* = 6). The *bar* represents LSD (0.05) for levels of abscisic acid (ABA), spermine, trehalose, and fructose

To examine if metabolic responses varied under drought stress, we selected the five most drought-tolerant genotypes (top 10 % genotypes) and the five most drought-sensitive genotypes (bottom 10 % genotypes) for metabolite profiling (Table 5). The levels of 14 metabolites, including ABA, JA, JA-Ile, betaine, proline, putrescine, spermine, spermidine, fructose, glucose, inositol, sucrose, trehalose, and raffinose were analyzed. Among the 14 metabolites, differences in ABA, spermine, trehalose, and fructose were found between the five best drought-tolerant genotypes and the five most drought-sensitive genotypes (Fig. 5). In general, the five best drought-tolerant genotypes tended to accumulate higher levels of ABA, spermine, trehalose, and fructose under drought stress than the five most drought-sensitive genotypes (Fig. 5).

## Discussion

### Evaluation of switchgrass germplasm with different physiological and metabolic parameters

In this study, we screened 49 diverse switchgrass genotypes for their tolerance to drought stress by measuring physiological, morphological, and metabolic traits. Our results indicate that the physiological parameters contributed more than the morphological traits in separating the control and drought-treated groups. This suggests that physiological characteristics may be closely associated with short-term drought tolerance in switchgrass. Previously, the evaluation of plant drought tolerance has been complicated due to inconsistencies in testing environments, the interactions between different developmental stages of plant growth, and the handling of a large number of plant genotypes [45]. Thus, no comprehensive, standardized system for measuring drought resistance has been established [46]. Indices that are based on yield loss under drought conditions, in comparison with normal conditions, have been used in crop breeding programs; however, these indices are labor intensive and time consuming [47, 48]. In this study, we measured a set of physiological parameters under drought treatment to effectively classify a relatively large collection of switchgrass germplasm. This process is non-destructive and sensitive to in planta conditions, which makes it favorable for collecting more reliable drought-related data.

Drought stress significantly altered the physiological parameters (Pn, *g*_s_, Tr, Ci, and WUE) of all 49 switchgrass genotypes (Additional file 3: Figure S2; Additional file 4: Figure S3; Additional file 5: Figure S4; Additional file 6: Figure S5; Additional file 7: Figure S6; Additional file 8: Figure S7; Additional file 9: Figure S8). During drought conditions, the Pn may be inhibited due to stomatal closure. This could inhibit RuBisco activity and increase respiration rates, ultimately leading to depleted carbohydrate reserves, reduced growth rates, and early plant senescence. In response to water deficit, the stomata may also close to conserve water; however, stomatal closure (lower *g*_s_) may block gas exchange and result in an increase in the O_2_/CO_2_ ratio. With an increase in excess O_2_ molecules, energy may be directed to them and the production of toxic reactive oxygen species (ROS) becomes a concern [49, 50]. In turn, these ROS may destroy important cellular components such as proteins, lipids, and nucleic acids, resulting in cell membrane damage (increased EL). Excess ROS may also damage components of the photosynthesis system, reducing the Pn and leaf RWC (Additional file 7: Figure S6) and increasing EL (Additional file 9: Figure S8) [51]. In order to cope with abiotic stresses, such as drought, plants have evolved the ability to evoke antioxidant defense systems, osmotic adjustments, and hormonal regulations of stomatal functions [17, 18].

The DSIs for each physiological parameter (Additional file 3: Figure S2; Additional file 4: Figure S3; Additional file 5: Figure S4; Additional file 6: Figure S5; Additional file 7: Figure S6; Additional file 8: Figure S7; Additional file 9: Figure S8) were used to evaluate the relative drought tolerance of all 49 switchgrass genotypes. Our results showed that the drought-tolerant genotypes had higher RWC, *g*_s_, Pn, Tr, and Ci, and a lower EL than the drought-sensitive genotypes. In addition, the five best drought-tolerant genotypes tended to accumulate higher levels of ABA, spermine, fructose, and trehalose under drought stress than the five most drought-sensitive genotypes (Fig. 5). It has been well documented that ABA induces stomatal closure and reduces water loss through transpiration [23]. Our results show that the drought-tolerant genotypes had higher levels of ABA, *g*_s_, and Tr, suggesting that these genotypes may achieve a higher tolerance to drought stress by maintaining better gas exchange (high *g*_s_ and Tr). This high level of gas exchange would reduce ROS toxicity, providing stronger signal-mediated regulations (Fig. 5, higher ABA, spermine, and trehalose) and osmotic adjustments (higher levels of trehalose and fructose). Fructose and trehalose are important osmoprotectants that facilitate osmotic adjustment. A previous study in Arabidopsis also showed that spermine is closely correlated with drought tolerance [52]. No consistent differences in any of the other metabolites between drought-tolerant and drought-sensitive genotypes were found in our study. In addition to regulating gas exchange, plants possess various antioxidant metabolites and enzymes to remove ROS. Stomatal functions and photosynthesis efficiency rates under drought conditions may not only be regulated by hormones, such as ABA. The integrity of these processes could also be maintained by other metabolites that might facilitate such harsh osmotic adjustments, ultimately improving drought tolerance in plants [23, 53].

Drought-tolerant genotypes have also been shown to have a higher photosynthetic function (higher Pn) relative to drought-sensitive genotypes. Similar results were also found in switchgrass [4], maize [54], and creeping bentgrass [55] under drought treatment. Mohamed [11] found that water stress affected several switchgrass cultivars (Carthage, Alamo, Kanlow, Southlow, Cave-in-Rock, Forestburg, Blackwell, Nebraska 28, Shelter, Shawnee, Dacotah, Sunburst, and WI) physiologically by decreasing photosynthesis. Jiang et al. [4] evaluated the upland switchgrass cultivar Cave-in-Rock and noted that drought stress reduced tissue water content, leaf dry weight, and chlorophyll fluorescence but increased total carotenoid concentration and EL.

### Methods for analysis of large physiological datasets

It is still challenging to reliably analyze and interpret large physiological datasets collected from plants grown under drought and well-watered conditions. Various methods and statistical models have been proposed for such analyses. Correlation analysis, PCA, and clustering are considered to be good methods for evaluating the relationships between the parameters and their principal components in phenotypic screening for drought tolerance [21, 47, 48]. In this study, PCA and correlation analysis showed that the differences in drought tolerance among the 49 switchgrass genotypes were largely due to variations in physiological parameters, especially Pn (Table 4; Fig. 4). Our results also found that some lowland genotypes, such as TEM-SEC, TEM-LoDorm, BN-13645-64, Alamo, and TEM-SLC, have relatively good tolerance to drought. These genotypes maintain higher Pn, Tr, g_s_, Ci, and RWC and lower EL in comparison to the upland genotypes (Additional file 3: Figure S2; Additional file 4: Figure S3; Additional file 5: Figure S4; Additional file 6: Figure S5; Additional file 7: Figure S6; Additional file 9: Figure S8; Table 5). Although the upland genotypes had a comparatively greater WUE than the lowland genotypes, our data showed that WUE was highly variable (Additional file 8: Figure S7). In fact, WUE alone may not be enough of a factor for evaluating drought tolerance [56].

A heatmap is a visual method that can be used to explore complex associations between multiple parameters collected from various treatments. It is often useful to combine heatmap with hierarchical clustering, which is a way of arranging items in a hierarchy based on the distance or similarity between them. Despite its benefits, heatmap analysis (Fig. 2) could not clearly identify the significant differences between the genotypes in this study. PCA biplots (Figs. 3, 4), however, could show the relative contributions of the parameters to the clustered groups. In our study, the PCA based on the DSI of seven physiological parameters yielded three PCs that accounted for 87.53 % of the total variance (Fig. 4; Additional file 10: Figure S9). For the purpose of evaluating switchgrass tolerance to drought stress, the three PCs were sufficient to represent the seven physiological parameters. To comprehensively evaluate the relative drought tolerance of the 49 switchgrass genotypes, a ranking value was calculated for each of the genotypes analyzed in this study (Table 5). Based on their ranking values, genotypes TEM-SEC, TEM-LoDorm, BN-13645-64, Alamo, BN-10860-61, BN-12323-69, TEM-SLC, T-2086, T-2100, T-2101, Caddo, and Blackwell-1 were more tolerant to drought stress. In contrast, genotypes Grif Nebraska 28, Grenville-2, Central Iowa Germplasm, Cave-in-Rock, Dacotah, and Nebraska 28 were relatively sensitive to drought stress.

## Conclusion

There is wide variation in the drought tolerance of the 49 switchgrass genotypes examined in this study. Based on DSI values for each physiological parameter, cluster analysis, and PCA ranking, we found that genotypes TEM-SEC, TEM-LoDorm, BN-13645-64, Alamo, BN-10860-61, BN-12323-69, TEM-SLC, T-2086, T-2100, T-2101, Caddo, and Blackwell-1 were more drought tolerant. We also found that genotypes Grif Nebraska 28, Grenville-2, Central Iowa Germplasm, Cave-in-Rock, Dacotah, and Nebraska 28 were relatively sensitive to drought stress. The physiological measurements and metabolic profiles generated in this study offered a sensitive, reliable approach for identifying switchgrass genotypes that are tolerant or sensitive to drought stress. The results of this study provide a foundation for further investigating the molecular mechanisms underlying switchgrass tolerance to drought stress.

## Methods

### Plant materials and culture

This study was performed in a greenhouse at Virginia Tech (Blacksburg, VA, USA). Diverse switchgrass germplasm accessions were originally obtained from the United States Department of Agriculture Germplasm Center and were maintained in the Virginia Tech Kentland Farm Agricultural Station (Blacksburg, VA, USA). One genotype from each of the 49 switchgrass accessions was chosen for this study (Table 1). Each switchgrass genotype was propagated by splitting tillers. On May 12, 2012, a tiller from each genotype was planted in a large pot (40 cm diam., 45 cm deep) filled with 12 kg of a mixture of sandy loam top soil and sand (2:1, v/v, 0.1–1.0 mm diam.). After 2 months of culture, six tillers from each genotype were transplanted into six plastic pots (17 cm diam., 20 cm high, with four holes at the bottom for drainage) and filled with 3.5 kg of a soil and sand mixture (soil:sand = 2:1 v/v, sand: 0.1–1.0 mm diam.). Of the 49 genotypes (Table 1), ten (AM-314/MS-155, BN-13645-64, BN-11357-63, Alamo, TEM-SEC, TEM-SLC, TEM-LoDorm, T-2086, BN-12323-69, and Kanlow) were lowland ecotypes [8, 57], and the rest were upland ecotypes.

The plants were grown in the greenhouse at temperatures of 30 ± 1 °C/25 ± 1 °C (day/night), a 14-h photoperiod, 75 % relative humidity, and with photosynthetically active radiation of approximately 500 μmol m^−2^s^−1^ (natural daylight supplemented with fluorescent lamps). The plants were irrigated daily, and fertilizer containing N (Bulldog brand, 28-8-18, 1 % ammonia N, 4.8 % nitrate N, and 22.2 % urea N; SQM North America, Atlanta, GA, USA) and micronutrients was applied at 0.49 kg m^−2^ every week.

### Drought stress treatment

After the plants were grown for 2 months (Sep 10, 2012) and had reached the E5 developmental stage [58], they were exposed to one of two soil moisture treatments (well-watered or drought stress) for 30 days.

The plants from each genotype were randomly assigned to either the control group (*n* = 6), which was kept well watered to maintain the soil moisture content at container capacity, or to the drought treatment group (*n* = 6), in which the soil moisture was allowed to gradually decline from day 0 to day 30 by reducing the amount of water used for irrigation. Water was added daily to compensate for 30–50 % ET loss during the experiment over the 30-day period. ET was determined by weighing the pots [59]. In addition, the volumetric soil moisture content (VWC) was monitored using a soil moisture meter (model HH2, Delta-T Devices, Cambridge, England).

In a separate experiment, the soil water content (SWC) of the growth media was determined based on differences in soil sample weight before and after drying at 105 °C to a constant weight. This difference was expressed as the percentage of the weight lost relative to the oven-dried weight. Soil samples were taken at different time points and each data point is the average of the measurements.

On average, the volumetric water content (VWC) was reduced from 40.00 to 21.24 % and the SWC was reduced from 26.21 to 17.34 % between days 0 and 15. Between days 15 and 25, the VWC was reduced from 21.24 to 10.94 % and the SWC was reduced from 17.34 to 8.09 %. Finally, the VWC was reduced from 10.94 to 5.79 % and the SWC was reduced from 8.09 to 4.31 % between days 25 and 30. The well-watered pots were irrigated daily to maintain approximately 40.0 % volumetric soil moisture (Table 6). The amount of water given each day was determined according to ET [59].

**Table 6:** The change in soil volumetric water content and soil water content over time in well-watered and drought conditions

Day	well-watered			Drought		
	ET (%)	VWC (%)	SWC (%)	ET (%)	VWC (%)	SWC (%)
0	100	40.2 ± 1.70	26.3 ± 1.04	100	40.0 ± 2.23	26.2 ± 2.40
5	100	39.8 ± 1.35	27.1 ± 1.82	50	34.7 ± 1.72	25.1 ± 2.90
10	100	40.4 ± 1.85	26.3 ± 1.48	50	28.5 ± 1.64	20.9 ± 2.94
15	100	40.3 ± 0.7	28.3 ± 0.61	40	21.2 ± 3.68	17.3 ± 1.66
20	100	38.6 ± 1.2	25.7 ± 1.57	40	14.2 ± 1.07	10.9 ± 2.18
25	100	40.2 ± 1.8	26.3 ± 1.61	30	10.9 ± 1.77	8.09 ± 3.57
30	100	41.6 ± 2.1	29.7 ± 1.17	30	5.8 ± 2.01	4.31 ± 2.53

VWC: volumetric water content; SWC: soil water content

*n* = 294 for VWC and *n* = 16 for SWC

### Physiological measurements

To measure electrolyte leakage (EL) and relative water content (RWC), leaf samples were collected after 0, 5, 10, 15, 20, 25, and 30 days of drought stress. At the same time points, the photosynthetic rate (Pn), stomatal conductance (*g*_s_), intercellular CO_2_ concentration (Ci), and transpiration rate (Tr) were determined. At the end of the experiment (30 days), leaf tissue samples for metabolite and genetic diversity analyses were collected and frozen in liquid N_2_.

Leaf electrolyte leakage (EL) was measured according to the method of Marcum [60] with some modifications. The top 2nd or 3rd mature leaf blades were excised and cut into 2-cm segments. After rinsing 3 times with deionized H_2_O, 0.2 g of the leaf tissue was placed in a 50-mL test tube containing 20 mL deionized H_2_O. The test tubes were agitated on a shaker for approximately 24 h, and the solution conductivity (*C*_1_) was measured with a conductivity meter (SR60IC, VWR, Radnor, PA, USA). The leaf samples were then autoclaved at 120 °C for 30 min, and when the tubes cooled to room temperature, the conductivity of the solution containing the killed tissue was measured (*C*_2_). The relative EL was calculated using the formula: EL (%) = (*C*_1_/*C*_2_) × 100.

Leaf relative water content (RWC) was determined according to the method of Barrs and Weatherley and was based on the following formula: RWC = (FW − DW)/(TW − DW) × 100, where FW is leaf fresh weight, DW (dry weight) is the weight of the leaves after drying at 85 °C for 3 days, and TW (turgid weight) is the weight of the leaves after soaking them in distilled water for 24 h at 20 °C.

The photosynthetic rate (Pn), stomatal conductance (*g*_s_), intercellular CO_2_ concentration (C_i_) and transpiration rate (Tr) were measured using a portable photosynthesis system (Li-6400XT, LI-COR, Inc., Lincoln, NE, USA) under a controlled atmosphere (385 μmol mol^−1^ CO_2_, 500 μmol s^−1^ flow rate, 26 °C) and a LI-COR 6400 LED external light source that provided a photosynthetic photon flux density (PPFD) of 2000 μmol m^−2^ s^−1^. The uppermost fully expanded leaf on the main tiller in each pot was selected for these measurements. Three readings were collected for each sample, and the average was used for statistical analysis.

### Metabolite extraction and derivatization

We analyzed a total of 14 drought stress tolerance-related metabolites [53, 61, 62], including ABA, JA, JA-Ile, betaine, proline, putrescine, spermine, spermidine, fructose, glucose, inositol, sucrose, trehalose, and raffinose.

Plant tissues were frozen in liquid nitrogen, lyophilized overnight and transferred to 2-mL screw-cap tubes (http://www.sarstedt.com) containing three 3.2-mm stainless steel beads (http://www.biospec.com). The tissue was ground, and aliquots of approximately 50 mg were automatically transferred to new tubes using the iWall instrument at the GLBRC Cell Wall facility at Michigan State University (https://www.glbrc.org/research/enabling-technologies). Chemical extraction was performed using a 10 % methanol and 1 % acetic acid solvent containing internal standards of 10 μM dh-JA, 10 μM ribitol, 10 μM [^2^H_3_]proline, and 1 μM [^2^H_6_]ABA. For extraction, 400 μL of extraction solvent was added to approximately 50 mg of ground plant tissue, and the mixture was incubated at 70 °C for 30 min. The extract was centrifuged for 15 min at 13,000 rpm, and 200 μL of supernatant was transferred to a new 1.5-mL microcentrifuge tube. For each plant, 60 μL of extract was transferred to each of two 96-well PCR tubes for the analysis of two groups of metabolites: (1) ABA, JA, and JA-Ile, and (2) polyamines and proline. For sugar analysis using GC–MS, 10 μL of the extract was transferred to a new 1.5-ml microcentrifuge tube for derivatization. For derivatization, 10 μL of extract was evaporated to complete dryness overnight using a SpeedVac. Ten microliters of 40 mg mL^−1^*O*-methylhydroxylamine hydrochloride in pyridine was added to the dried plant extract and the tubes incubated for 90 min at 30 °C with gentle rocking. Forty-five microliters of *N*-methyl-*N*-trimethylsilyltrifluoroacetamide (MSTFA) with 1 % trimethylchlorosilane (TMCS) was then added to the mixture, and the tubes incubated at 37 °C for an additional 30 min. For sugar analysis, 50 μL of the derivatized product was transferred to a glass vial containing a glass insert (http://www.restek.com). All materials were barcoded to keep track of each sample throughout the entire extraction and derivatization procedure.

### Metabolite determination using GC–MS

To analyze the plants’ sugar profiles, GC–MS was performed using a 6890N network GC system with a 5973 mass selective detector (Agilent Technologies, http://www.agilent.com, Santa Clara, CA, USA). Separation was achieved by injection of 1 μL of derivatized extract into a 30-m VF-5 MS column (30 m × 0.25 mm × 0.25 μM; Agilent), with a 10-m EZ-Guard (Agilent), using the following temperature profile: 80 °C for 1 min; 40 °C min^−1^ to 220 °C for 3 min; 40 °C min^−1^ to 320 °C for 6 min; and 270 °C for 1 min. Sugar metabolites were detected using selected ion monitoring (SIM) mode with m/z values of 205.2, 217.0, 217.2, 305.0, 307.2, 319.2, 319.3, and 361.3 throughout the GC–MS run.

### Metabolite analysis using LC–MS/MS

For the analysis of ABA, JA, and JA-Ile, methods from Chung et al. [63] were used with modifications. Briefly, extracts (10 μL) were injected into an Ascentis Express C18 column (2.7 μM, 2.1 × 50 mm, Supelco Analytical) and attached to an Acquity Ultraperformance Liquid Chromatography System (Waters, http://www.waters.com, Milford, MA, USA) for LC reverse-phase analysis. The column temperature was maintained at 50 °C. A steep gradient was executed between solvents A and B (A—0.15 % formic acid in MilliQ water, B—methanol) with an analysis time of 3 min/sample and a 0.4 mL min^−1^ flow rate. The gradient profile was as follows: 30 % B for the initial step; a linear gradient to 70 % B in 1.5 min; 100 % B in 2 min; 100 % B maintained for 2.5 min; and 30 % B from 2.5 min to 3 min. Mass spectra were acquired using electrospray ionization in negative ion mode and multiple reaction monitoring (MRM). A Quattro Premier XE tandem quadrupole mass spectrometer (Waters) was coupled to the LC to identify and detect analytic signals under the following conditions: 3.00 kV capillary voltage; 100 °C source temperature; 300 °C desolvation temperature; 20 L h^−1^ nebulizer nitrogen flow rate; and 300 L h^−1^ desolvation nitrogen gas flow rate. The transitions from precursor molecules to characteristic product ions were monitored for JA (*m*/*z* 209 > 59), dh-JA (*m*/*z* 211 > 59), JA-Ile (*m*/*z* 322 > 130), ABA (*m*/*z* 263 > 153), and [^2^H_6_] ABA (*m*/*z* 269 > 159). The collision energies and source cone potentials were optimized for each transition using Waters QuanOptimize software. Because this method does not distinguish JA-Ile from JA-Leu, the values reported for JA-Ile represent the sum of JA-Ile and JA-Leu. In Arabidopsis seedlings, the amount of JA-Leu is reported to be <25 % that of JA-Ile [64].

For the analysis of putrescine and spermine, methods from Gu et al. [65] were used with modifications. Briefly, the extracts (10 μL) were injected into a Symmetry C18 column (2.1 × 100 mm, 3.5 μM particle size, Waters) and attached to a Shimadzu (Columbia, MD, USA) LC-20AD HPLC system for LC reverse-phase analysis. The column temperature was maintained at 30 °C. A steep gradient was executed between solvents A and B (A—1 mM perfluoroheptanoic acid in MilliQ water, B—acetonitrile) with an analysis time of 6 min/sample and a 0.3 mL min^−1^ flow rate. The gradient profile was as follows: 2 % B for the initial step; a linear gradient to 20 % B in 0.1 min; 80 % B in 2.5 min; 80 % B maintained for 4 min; 20 % B in 4.1 min; and 2 % B in 6 min. Mass spectra were acquired using electrospray ionization in positive ion mode and MRM. A Quattro micro mass spectrometer (Waters) was coupled to the LC to identify and detect analytic signals under the following conditions: electrospray negative ionization mode; 3.17 kV capillary voltage; 110 °C source temperature; 350 °C desolvation temperature; 20 L h^−1^ nebulizer nitrogen flow rate; and 400 L h^−1^ desolvation nitrogen gas flow rate. The transitions from precursor molecules to characteristic product ions were monitored for putrescine (*m*/*z* 89 > 72), proline (*m*/*z* 116 > 70), betaine (*m*/*z* 118 > 59), [^2^H_3_]proline (*m*/*z* 119 > 73), spermidine (*m*/*z* 146 > 72), and spermine (203 > 112). The collision energies and source cone potentials were optimized for each transition using Waters QuanOptimize software.

### Drought tolerance evaluation

To assess the drought tolerance of different populations, the drought stress index (DSI) was used in this study. DSI was calculated using the formula: DSI = (value of trait under stress condition)/(value of trait under controlled condition) × 100 [15].

To assess the drought tolerance of different genotypes, the PCA ranking value was used in this study. The PCA ranking value for each switchgrass genotype was calculated using the formula:

Ranking value = (contribution of PC1 (%) × PC1) + (contribution of PC2 (%) × PC2) + (contribution of PC3 (%) × PC3) [44].

### DNA extraction and genetic diversity analysis

DNA was extracted from approximately 200 mg of leaf tissue from each of the 49 genotypes using the CTAB method [66]. The quality of the DNA was assessed by electrophoresis on 0.8 % agarose gels, and the quantity of the DNA was measured by comparing the samples to standardized lambda DNA size markers.

For SRAP-PCR amplification, 12 pairs of previously reported SRAP primers were selected for this study (Table 2) [67]. SRAP analysis was performed as described previously [40]. Briefly, each 20 μL PCR reaction mixture consisted of 40 ng genomic DNA, 0.2 mM dNTPs, 2.5 mM MgCl_2_, 0.5 μM primers, 1× PCR buffer, and 1 unit of Taq polymerase. The amplification was performed in four steps: pre-denaturation at 94 °C for 4 min; 5 cycles of 1 min denaturation at 94 °C, 1 min annealing at 35 °C and 1.5 min extension at 72 °C; 35 cycles of 1 min at 94 °C, 1 min at 50 °C, and 1.5 min at 72 °C; and a final extension step at 72 °C for 7 min. The PCR fragments were separated on a 5 % agarose gel, stained with 0.01 % ethidium bromide, and visualized using a Gel-Document Image System™ under UV light (Bio-Rad, Hercules, CA, USA).

### Experimental design and statistical analysis

A split plot design was used in this experiment, with the soil moisture regimes as the main plots and the switchgrass genotypes as the subplots. Each genotype had six replicates for each soil moisture treatment (well-watered and drought). All data were subjected to analysis of variance (ANOVA, SAS 8.1, SAS Institute Inc., Cary, NC, USA). The treatment means were separated using Fisher’s protected least significant difference (LSD) test at a 5 % probability level.

R statistical software (PCA analysis, R2.15.1 by R Development Core Team) was used to determine the correlations between physiological and morphological traits and to perform principal component analysis of the traits.

Each genomic DNA fragment obtained from the SRAP primer combinations was scored as present (1) or absent (0). These data were used to calculate genetic distances and to draw genetic distance dendrograms of the 49 switchgrass genotypes. The dendrograms were drawn using NTSYS-pc version 2.2 and were based on the DICE matrix and UPGMA (Unweighted Pair Group Method) arithmetical averages in the SAHN module. Concordance between the genotypic data and the dendrogram was determined using a Mantel test [68]. In addition, genetic distance dendrograms were drawn using DARwin5 software, which is based on the DICE matrix and UPGMA in the hierarchical clustering module and on unweighted neighbor-joining in the neighbor-joining module.

## Additional files

**Additional file 1: Figure S1.** Principal component analysis biplot of morphological and physiological traits of 49 switchgrass genotypes under well-watered and drought stress conditions after 15 days of treatment. The seven physiological parameters (Pn, Ci, *g*_s_, Tr, RWC, WUE and EL) allow to separate 49 switchgrass genotypes that were either grown under well-watered (circled) or drought treatment (box) conditions. Arrows represent physiological traits with various length based on the impact of each trait on the separation of genotypes. RWC: relative water content; EL: electrolyte leakage; Pn: photosynthetic rate; g_s_: stomatal conductance; Tr: transpiration rate; Ci: intercellular CO_2_ concentration; WUE: water use efficiency. LL: leaf length; LW: leaf width; SL: leaf sheath length. Group A: well-watered; Group B: drought treatment.
**Additional file 2.** Effect of drought stress on morphological parameters at 30 d of experiment (*n* = 6), and effect of drought stress on morphological parameters of lowland and upland lines at 30 d of experiment, (*n* = 294). LL: leaf length; LW: leaf width; SL: leaf sheath length; SE: standard error.
**Additional file 3: Figure S2.** The photosynthetic rate (Pn) of 49 genotypes under well-watered conditions (control, solid bar) or drought stress (open bar) after 30 days of treatment. The genotype represents the DSI. The greater the DSI, the better the drought tolerance. The values are the means (*n* = 6).The bar represents LSD (0.05) for DSI.
**Additional file 4: Figure S3.** The stomatal conductance (*g*_s_) of 49 genotypes under well-watered conditions (control, solid bar) or drought stress (open bar) after 30 days of treatment. The genotype represents the DSI. The greater the DSI, the better the drought tolerance. The values are the means (*n* = 6). The bar represents LSD (0.05) for DSI.
**Additional file 5: Figure S4.** The transpiration rate (Tr) of 49 genotypes under well-watered conditions (control, solid bar) or drought stress (open bar) after 30 days of treatment. The genotype represents the DSI. The greater the DSI, the better the drought tolerance. The values are the means (*n* = 6). The bar represents LSD (0.05) for DSI.
**Additional file 6: Figure S5** The intercellular CO_2_ concentration (Ci) of 49 genotypes under well-watered conditions (control, solid bar) or drought stress (open bar) after 30 days of treatment. The genotype represents the DSI. The greater the DSI, the better the drought tolerance. The values are the means (*n* = 6). The bar represents LSD (0.05) for DSI.
**Additional file 7: Figure S6.** The relative water content (RWC) of leaves from 49 genotypes under well-watered conditions (control, solid bar) or drought stress (open bar) after 30 days of treatment. The genotype represents the DSI. The greater the DSI, the better the drought tolerance. The values are the means of four replicates (*n* = 6). The bar represents LSD (0.05) for DSI.
**Additional file 8: Figure S7.** The water use efficiency (WUE) of 49 genotypes under well-watered conditions (control, solid bar) or drought stress (open bar) after 30 days of treatment. The genotype represents the DSI. The greater the DSI, the better the drought tolerance. The values are the means (*n* = 6). The bar represents LSD (0.05) for DSI.
**Additional file 9: Figure S8.** The leaf electrolyte leakage (EL) of 49 genotypes under well-watered conditions (control, solid bar) or drought stress (open bar) after 30 days of treatment. The genotype represents the DSI. The lower the DSI, the better the drought tolerance. The values are the means (*n* = 6). The bar represents LSD (0.05) for DSI.
**Additional file 10: Figure S9.** Proportion of variance for principal component analysis based on the DSI of seven physiological traits of 49 switchgrass genotypes under well-watered (control) and drought stress conditions after 30 days of treatment.

## Abbreviations

ET: evapotranspiration
RWC: relative water content
EL: electrolyte leakage
Pn: photosynthetic rate
Gs: stomatal conductance
Tr: transpiration
SRAP: sequence-related amplified polymorphism
Ci: intercellular CO_2_ concentration
WUE: water use efficiency
PCA: principal component analysis
ABA: abscisic acid
JA: jasmonic acid
JA-Ile: jasmonic acid–isoleucine
GB: glycine betaine
PAs: polyamines
LL: leaf length
LW: leaf width
SL: leaf sheath length
