# Supplementary figures and images for: Assessment of drought tolerance of 49 switchgrass (Panicum virgatum) genotypes using physiological and morphological parameters
Source: Biotechnol Biofuels. 2015 Sep 22;8:152. doi: 10.1186/s13068-015-0342-8 (PMC4578271; doi:10.1186/s13068-015-0342-8)

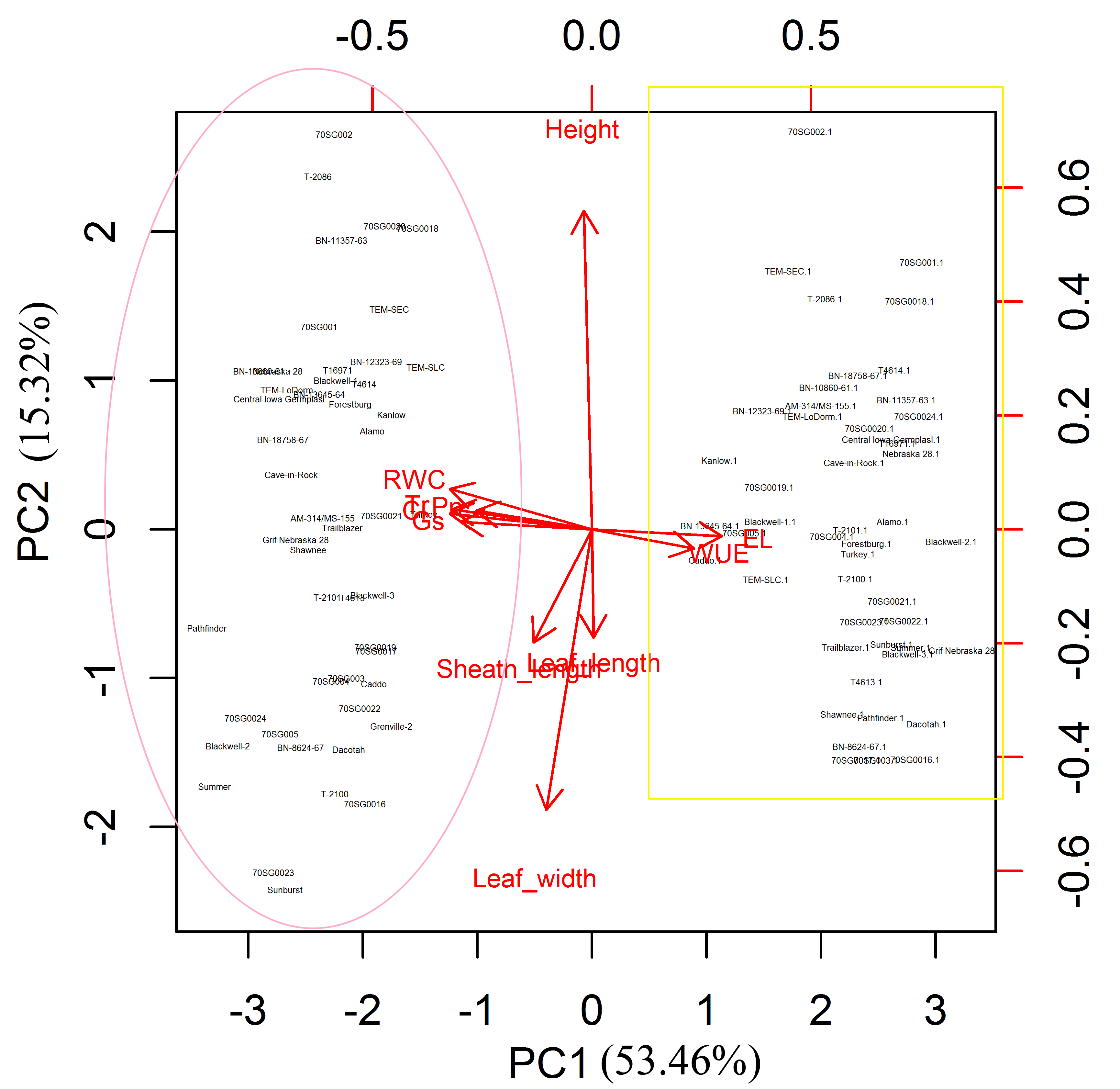

Supplement: Supplementary file 1 — Additional file 1: Figure S1. Principal component analysis biplot of morphological and physiological traits of 49 switchgrass genotypes under well-watered and drought stress conditions after 15 days of treatment. The seven physiological parameters (Pn, Ci, g s, Tr, RWC, WUE and EL) allow to separate 49 switchgrass genotypes that were either grown under well-watered (circled) or drought treatment (box) conditions. Arrows represent physiological traits with various length based on the impact of each trait on the separation of genotypes. RWC: relative water content; EL: electrolyte leakage; Pn: photosynthetic rate; gs: stomatal conductance; Tr: transpiration rate; Ci: intercellular CO2 concentration; WUE: water use efficiency. LL: leaf length; LW: leaf width; SL: leaf sheath length. Group A: well-watered; Group B: drought treatment. [file 13068_2015_342_MOESM1_ESM.tif]

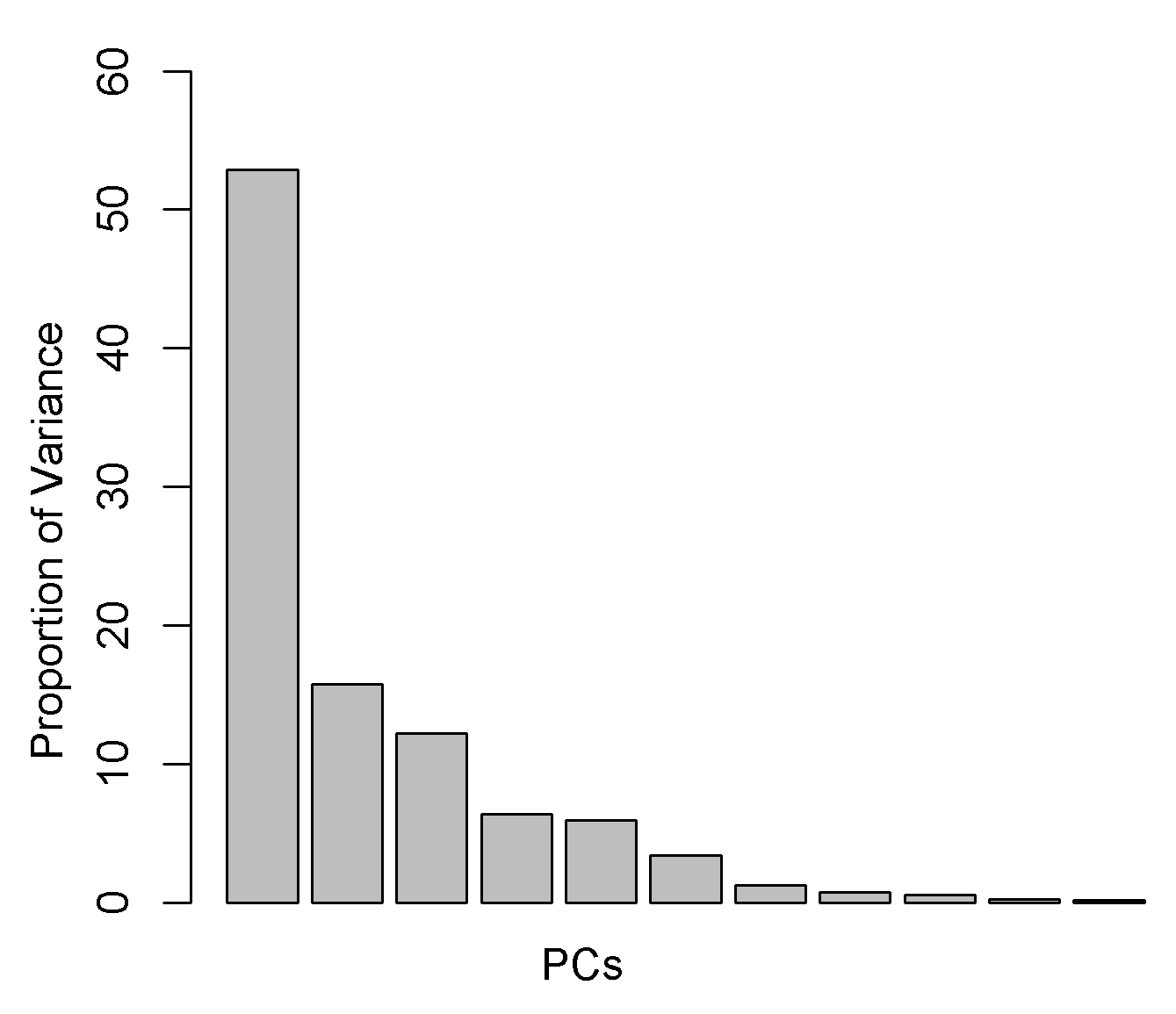

Supplement: Supplementary file 10 — Additional file 10: Figure S9. Proportion of variance for principal component analysis based on the DSI of seven physiological traits of 49 switchgrass genotypes under well-watered (control) and drought stress conditions after 30 days of treatment. [file 13068_2015_342_MOESM10_ESM.tif]
