# Supplementary material for: Assessment of drought tolerance of 49 switchgrass (Panicum virgatum) genotypes using physiological and morphological parameters
Source: Biotechnol Biofuels. 2015 Sep 22;8:152. doi: 10.1186/s13068-015-0342-8 (PMC4578271; doi:10.1186/s13068-015-0342-8)
Supplement: Supplementary file 2 — Additional file 2. Effect of drought stress on morphological parameters at 30 d of experiment (n = 6), and effect of drought stress on morphological parameters of lowland and upland lines at 30 d of experiment, (n = 294). LL: leaf length; LW: leaf width; SL: leaf sheath length; SE: standard error. [file 13068_2015_342_MOESM2_ESM.docx]

**Supplemental table 1. Effect of drought stress on morphological parameters at 30 d of experiment (n=6), LL: leaf length; LW: leaf width; SL: leaf sheath length; SE: standard error.**

| **Ecotypes** | **Lines** | **Well-watered** | | | | | | | | **Drought** | | | | | | | |
| --- | --- | --- | --- | --- | --- | --- | --- | --- | --- | --- | --- | --- | --- | --- | --- | --- | --- |
|  |  | **Height (cm)** | **SE** | **LL (cm)** | **SE** | **LW (cm)** | **SE** | **SL (cm)** | **SE** | **Height (cm)** | **SE** | **LL (cm)** | **SE** | **LW (cm)** | **SE** | **SL (cm)** | **SE** |
| Lowland | TEM-SLC | 118.82 | 4.4608 | 58.11 | 3.663 | 1.614 | 0.103 | 16.65 | 2.137 | 96.31 | 4.5717 | 60.69 | 5.426 | 1.391 | 0.102 | 13.64 | 0.796 |
|  | TEM-SEC | 124.32 | 6.3750 | 42.86 | 3.266 | 1.832 | 0.036 | 20.11 | 4.054 | 115.01 | 4.6068 | 41.70 | 4.141 | 1.596 | 0.052 | 13.39 | 2.773 |
|  | TEM-LoDorm | 127.62 | 9.0514 | 49.50 | 4.600 | 1.760 | 0.152 | 20.97 | 1.056 | 113.26 | 4.6877 | 50.26 | 6.282 | 1.494 | 0.053 | 20.29 | 0.901 |
|  | T-2086 | 137.48 | 4.1643 | 50.49 | 2.437 | 1.647 | 0.030 | 18.29 | 3.628 | 93.91 | 3.8139 | 51.04 | 4.432 | 1.483 | 0.056 | 18.98 | 1.234 |
|  | Kanlow | 133.03 | 5.0461 | 51.29 | 2.035 | 1.109 | 0.051 | 19.35 | 2.125 | 102.68 | 4.8400 | 49.90 | 3.716 | 1.133 | 0.068 | 19.21 | 4.062 |
|  | BN-13645-64 | 128.94 | 6.2066 | 55.02 | 3.218 | 1.592 | 0.043 | 27.24 | 2.671 | 115.60 | 4.6412 | 57.47 | 4.978 | 1.396 | 0.039 | 14.46 | 0.782 |
|  | BN-12323-69 | 127.65 | 12.2653 | 35.42 | 2.142 | 1.293 | 0.043 | 15.67 | 1.986 | 137.71 | 13.0059 | 35.86 | 3.479 | 1.346 | 0.046 | 22.53 | 2.160 |
|  | BN-11357-63 | 124.74 | 5.0100 | 40.38 | 1.641 | 1.400 | 0.037 | 15.34 | 2.218 | 132.73 | 14.4534 | 39.29 | 2.960 | 1.333 | 0.040 | 14.36 | 1.571 |
|  | AM-314/MS-155 | 99.48 | 5.0422 | 55.60 | 2.587 | 1.452 | 0.042 | 20.11 | 0.914 | 95.53 | 10.9509 | 56.19 | 4.784 | 1.492 | 0.069 | 15.13 | 2.098 |
|  | Alamo | 106.20 | 7.7300 | 51.93 | 2.486 | 1.555 | 0.050 | 20.97 | 0.973 | 110.28 | 6.8659 | 50.51 | 3.972 | 1.576 | 0.050 | 13.50 | 0.672 |
| upland | Turkey | 44.29 | 7.9705 | 6.00 | 0.958 | 0.804 | 0.032 | 5.50 | 0.627 | 46.72 | 5.4451 | 6.26 | 0.681 | 0.800 | 0.019 | 4.03 | 0.546 |
|  | tral lowa Germplasl | 126.46 | 5.2962 | 37.66 | 1.838 | 0.989 | 0.037 | 24.86 | 4.661 | 129.93 | 6.3062 | 36.64 | 2.924 | 1.033 | 0.039 | 12.81 | 1.643 |
|  | Trailblazer | 49.86 | 10.1613 | 62.98 | 8.267 | 1.183 | 0.104 | 16.89 | 1.863 | 29.09 | 8.3479 | 45.46 | 3.593 | 1.214 | 0.087 | 21.34 | 1.376 |
|  | T4614 | 118.43 | 5.8306 | 50.15 | 3.292 | 0.813 | 0.037 | 22.25 | 1.035 | 143.33 | 8.0838 | 50.37 | 4.173 | 0.818 | 0.066 | 17.63 | 0.722 |
|  | T4613 | 42.03 | 5.9882 | 39.51 | 2.731 | 1.317 | 0.166 | 19.40 | 1.628 | 46.85 | 1.5740 | 39.24 | 1.587 | 1.168 | 0.034 | 16.13 | 2.361 |
|  | T-2101 | 64.34 | 16.0436 | 30.71 | 2.865 | 1.391 | 0.120 | 13.22 | 0.684 | 33.94 | 0.7334 | 33.11 | 1.588 | 1.368 | 0.063 | 15.43 | 0.675 |
|  | T-2100 | 47.66 | 13.6724 | 73.07 | 7.883 | 1.557 | 0.174 | 16.93 | 2.351 | 25.80 | 3.0857 | 54.42 | 3.540 | 1.313 | 0.024 | 20.19 | 0.442 |
|  | T16971 | 126.80 | 5.9428 | 31.73 | 1.529 | 0.868 | 0.077 | 31.26 | 2.664 | 123.96 | 5.3001 | 32.08 | 2.783 | 0.866 | 0.083 | 11.54 | 0.519 |
|  | Sunburst | 52.57 | 5.9686 | 50.57 | 5.394 | 1.330 | 0.178 | 8.68 | 1.799 | 39.07 | 4.2517 | 50.58 | 2.422 | 1.122 | 0.126 | 13.34 | 0.473 |
|  | Summer | 105.26 | 6.4294 | 65.61 | 6.537 | 1.653 | 0.041 | 18.78 | 1.884 | 84.33 | 9.3382 | 62.61 | 0.000 | 1.776 | 0.039 | 17.11 | 0.230 |
|  | Shawnee | 50.02 | 5.3446 | 56.59 | 5.872 | 1.313 | 0.096 | 19.64 | 1.622 | 40.99 | 8.4843 | 48.75 | 1.719 | 1.122 | 0.049 | 17.56 | 1.119 |
|  | Pathfinder | 55.25 | 6.4915 | 63.44 | 4.793 | 1.480 | 0.182 | 16.92 | 1.400 | 53.55 | 2.5647 | 50.94 | 2.283 | 1.076 | 0.032 | 16.67 | 0.889 |
|  | Nebraska 28 | 119.70 | 5.5265 | 45.77 | 2.379 | 1.215 | 0.081 | 22.00 | 2.564 | 139.84 | 6.4291 | 47.80 | 3.918 | 1.100 | 0.042 | 12.88 | 0.514 |
|  | Grif Nebraska 28 | 64.78 | 14.5990 | 44.13 | 3.718 | 1.026 | 0.093 | 11.86 | 1.703 | 37.84 | 0.5475 | 36.59 | 2.265 | 1.098 | 0.051 | 9.74 | 0.153 |
|  | Genville-2 | 56.93 | 2.9288 | 57.03 | 1.287 | 1.567 | 0.120 | 23.65 | 0.882 | 44.01 | 0.7332 | 57.49 | 2.882 | 1.368 | 0.063 | 20.77 | 2.936 |
|  | Forestburg | 136.08 | 7.0173 | 45.58 | 2.219 | 0.615 | 0.193 | 9.48 | 1.364 | 99.48 | 25.4053 | 44.34 | 3.541 | 1.006 | 0.046 | 12.71 | 0.549 |
|  | Dacotah | 70.30 | 7.6703 | 48.41 | 2.693 | 1.677 | 0.073 | 14.58 | 0.997 | 47.02 | 2.2870 | 59.39 | 2.307 | 1.850 | 0.063 | 17.22 | 0.359 |
|  | Cave-in-rock | 113.18 | 7.7924 | 47.35 | 4.273 | 1.471 | 0.070 | 6.92 | 0.471 | 119.21 | 9.7972 | 47.45 | 4.723 | 1.280 | 0.076 | 16.96 | 0.992 |
|  | Caddo | 77.76 | 10.7505 | 37.75 | 2.527 | 1.524 | 0.150 | 13.10 | 0.936 | 72.13 | 13.4944 | 31.92 | 0.980 | 1.214 | 0.028 | 15.41 | 0.664 |
|  | BN-8624-67 | 71.63 | 3.7525 | 51.41 | 3.975 | 1.701 | 0.184 | 17.98 | 1.000 | 57.17 | 1.5317 | 40.57 | 2.107 | 1.161 | 0.014 | 20.01 | 0.657 |
|  | BN-18757-67 | 115.74 | 5.1859 | 47.33 | 7.328 | 1.302 | 0.059 | 22.36 | 1.661 | 100.96 | 4.0469 | 48.30 | 8.758 | 1.154 | 0.053 | 6.85 | 0.284 |
|  | BN-10860-61 | 120.65 | 6.7470 | 50.58 | 2.029 | 0.863 | 0.035 | 9.94 | 0.545 | 133.31 | 11.4141 | 49.20 | 3.676 | 0.917 | 0.036 | 10.06 | 0.681 |
|  | Blackwell-3 | 47.94 | 3.2584 | 45.08 | 4.558 | 1.410 | 0.133 | 17.08 | 1.691 | 33.34 | 2.3514 | 42.49 | 2.413 | 1.340 | 0.069 | 16.17 | 0.777 |
|  | Blackwell-2 | 43.95 | 10.6003 | 46.98 | 2.715 | 1.694 | 0.231 | 16.92 | 2.189 | 27.70 | 1.3716 | 52.24 | 2.932 | 1.151 | 0.063 | 19.30 | 1.683 |
|  | Blackwell-1 | 122.92 | 7.3225 | 33.60 | 1.352 | 0.885 | 0.052 | 21.29 | 2.634 | 111.65 | 7.4968 | 55.20 | 3.364 | 1.178 | 0.087 | 13.53 | 1.832 |
|  | 70SG005 | 56.90 | 8.0524 | 48.45 | 3.807 | 1.676 | 0.210 | 14.51 | 1.215 | 32.16 | 6.8309 | 54.49 | 3.236 | 1.198 | 0.053 | 17.27 | 1.149 |
|  | 70SG004 | 57.49 | 5.2845 | 46.23 | 4.907 | 1.402 | 0.185 | 17.14 | 1.486 | 35.23 | 0.9438 | 51.09 | 0.469 | 0.983 | 0.034 | 21.91 | 2.684 |
|  | 70SG003 | 57.45 | 13.0107 | 45.72 | 4.072 | 1.394 | 0.076 | 18.76 | 1.622 | 67.96 | 20.7944 | 49.24 | 2.845 | 1.434 | 0.058 | 19.85 | 0.907 |
|  | 70SG0024 | 47.68 | 7.0070 | 58.03 | 6.284 | 1.463 | 0.064 | 21.02 | 2.079 | 20.44 | 1.3802 | 40.61 | 3.280 | 0.595 | 0.026 | 18.48 | 0.077 |
|  | 70SG0023 | 57.39 | 5.2634 | 57.58 | 6.282 | 1.551 | 0.196 | 20.80 | 2.132 | 41.03 | 2.0206 | 50.74 | 4.373 | 1.248 | 0.039 | 17.46 | 2.224 |
|  | 70SG0022 | 63.64 | 10.8328 | 40.49 | 3.324 | 1.575 | 0.192 | 13.16 | 1.286 | 84.22 | 1.6389 | 44.13 | 0.484 | 1.179 | 0.012 | 18.10 | 1.031 |
|  | 70SG0021 | 115.52 | 7.8533 | 32.55 | 3.418 | 0.951 | 0.046 | 8.86 | 3.221 | 97.83 | 11.4104 | 31.66 | 3.902 | 0.919 | 0.044 | 10.56 | 0.692 |
|  | 70SG0020 | 149.99 | 8.3549 | 38.94 | 4.601 | 1.062 | 0.060 | 15.43 | 3.414 | 96.60 | 5.8669 | 37.89 | 5.113 | 1.002 | 0.053 | 14.77 | 1.637 |
|  | 70SG002 | 143.02 | 2.8949 | 38.74 | 2.704 | 0.962 | 0.041 | 18.81 | 0.781 | 126.56 | 5.1127 | 38.89 | 3.321 | 0.886 | 0.076 | 15.97 | 1.615 |
|  | 70SG0019 | 82.30 | 6.5649 | 53.03 | 4.720 | 1.669 | 0.190 | 19.34 | 1.530 | 69.07 | 2.8878 | 41.66 | 2.811 | 1.116 | 0.059 | 17.46 | 1.303 |
|  | 70SG0018 | 139.16 | 8.6876 | 47.58 | 7.153 | 0.813 | 0.064 | 17.28 | 3.139 | 143.74 | 6.8959 | 46.30 | 7.644 | 0.892 | 0.055 | 6.92 | 0.797 |
|  | 70SG0017 | 30.92 | 2.4048 | 60.10 | 2.154 | 1.261 | 0.113 | 15.68 | 0.994 | 16.89 | 2.2517 | 47.68 | 3.686 | 1.385 | 0.019 | 15.13 | 0.643 |
|  | 70SG0016 | 47.41 | 5.8133 | 70.51 | 4.272 | 1.301 | 0.135 | 18.52 | 1.337 | 49.11 | 0.7040 | 57.97 | 3.233 | 1.537 | 0.082 | 19.14 | 1.033 |
|  | 70SG001 | 106.77 | 4.6750 | 53.98 | 4.178 | 0.879 | 0.033 | 14.59 | 1.789 | 147.41 | 11.0852 | 56.38 | 5.652 | 0.857 | 0.032 | 14.97 | 0.730 |

**Supplemental table 2. Effect of drought stress on morphological parameters of lowland and upland lines at 30 d of experiment, (n=294)**

**LL: leaf length; LW: leaf width; SL: leaf sheath length.**

|  | **Well-watered** | | | | **Drought** | | | |
| --- | --- | --- | --- | --- | --- | --- | --- | --- |
|  | **Height (cm)** | **LL (cm)** | **LW (cm)** | **SL (cm)** | **Height (cm)** | **LL (cm)** | **LW (cm)** | **SL (cm)** |
| lowland | 122.83 ±3.72 | 49.06 ±2.30 | 1.525 ±0.069 | 19.47 ±1.088 | 111.30 ±4.77 | 49.29 ±2.56 | 1.424 ±0.043 | 16.55 ±1.063 |
| upland | 82.06 ±5.72 | 47.72 ±1.98 | 1.272 ±0.050 | 16.81 ±0.837 | 73.06 ±6.66 | 45.44 ±1.65 | 1.147 ±0.040 | 15.47 ±0.663 |
